# The long non-coding RNA Meg3 is dispensable for hematopoietic stem cells

**DOI:** 10.1038/s41598-019-38605-8

**Published:** 2019-02-14

**Authors:** Pia Sommerkamp, Simon Renders, Luisa Ladel, Agnes Hotz-Wagenblatt, Katharina Schönberger, Petra Zeisberger, Adriana Przybylla, Markus Sohn, Yunli Zhou, Anne Klibanski, Nina Cabezas-Wallscheid, Andreas Trumpp

**Affiliations:** 1 Division of Stem Cells and Cancer, German Cancer Research Center (DKFZ), 69120 Heidelberg, Germany; 2 Heidelberg Institute for Stem Cell Technology and Experimental Medicine (HI-STEM gGmbH), 69120 Heidelberg, Germany; 3 Faculty of Biosciences, Heidelberg University, 69117 Heidelberg, Germany; 4 Core Facility Omics IT and Data Management, German Cancer Research Center (DKFZ), 69120 Heidelberg, Germany; 5 Max Planck Institute of Immunobiology and Epigenetics, 79108 Freiburg, Germany; 6 Neuroendocrine Unit, Massachusetts General Hospital and Harvard Medical School, Boston, MA 02114 USA

## Abstract

The long non-coding RNA (lncRNA) Maternally Expressed Gene 3 (*Meg3*) is encoded within the imprinted *Dlk1-Meg3* gene locus and is only maternally expressed. *Meg3* has been shown to play an important role in the regulation of cellular proliferation and functions as a tumor suppressor in numerous tissues. *Meg3* is highly expressed in mouse adult hematopoietic stem cells (HSCs) and strongly down-regulated in early progenitors. To address its functional role in HSCs, we used MxCre to conditionally delete *Meg3* in the adult bone marrow of Meg3^mat-flox/pat-wt^ mice. We performed extensive *in vitro* and *in vivo* analyses of mice carrying a *Meg3* deficient blood system, but neither observed impaired hematopoiesis during homeostatic conditions nor upon serial transplantation. Furthermore, we analyzed VavCre Meg3^mat-flox/pat-wt^ mice, in which *Meg3* was deleted in the embryonic hematopoietic system and unexpectedly this did neither generate any hematopoietic defects. In response to interferon-mediated stimulation, *Meg3* deficient adult HSCs responded highly similar compared to controls. Taken together, we report the finding, that the highly expressed imprinted lncRNA *Meg3* is dispensable for the function of HSCs during homeostasis and in response to stress mediators as well as for serial reconstitution of the blood system *in vivo*.

## Introduction

Hematopoietic stem cells (HSCs) are characterized by their unique self-renewal capacity and multipotency^1^. As most mature blood cells are relatively short-lived, there is a constant need for the replenishment of functional effector cells. Along a hematopoietic cascade, HSCs first give rise to multipotent progenitor populations (MPPs) which differentiate into committed progenitors and will eventually give rise to mature effector cells^2^. In homeostasis, HSCs are characterized by a deeply quiescent cell status, called dormancy, protecting them from DNA damage and preserving their genomic integrity^2,3^. During stress response, such as upon infection and inflammation, HSCs exit the G_0_ dormant state and enter the cell division cycle^4,5^. The unique characteristics and the potential of HSCs have long been known^2,6^, however, only recently molecular expression signatures, signaling pathways and cell surface markers were identified by state-of-the-art OMICs analyses on a population and at the single-cell level^7–9^. Especially genes that show a very high and specific expression in HSCs are of major interest, as these genes are potential candidates involved in controlling HSC function, self-renewal and multipotency. Besides protein-coding genes, multiple long non-coding RNAs (lncRNAs, >200 nucleotides) were identified to be expressed in hematopoietic stem/progenitors and are known to play functional roles during normal and malignant hematopoiesis such as Lnc-HSC-2 and Xist^10–12^. One of the genes we and others identified as very highly and specifically expressed in HSCs is the lncRNA Maternally Expressed Gene 3 (*Meg3*)^8,9,13^.

*Meg3*, also known as gene trap locus 2 (*Gtl2*), is a lncRNA encoded in the imprinted *Dlk1-Meg3* gene locus^14,15^. The cis-elements regulating *Meg3* expression consist of two differentially methylated regions (DMRs), intergenic (IG)-DMR and Meg3-DMR, respectively^16^. *Meg3*, together with multiple non-coding RNAs and small non-coding RNAs, including the largest mammalian miRNA mega-cluster, is expressed from the maternally inherited allele only. IG-DMR and Meg3-DMR are unmethylated on the maternal allele, whereas they are methylated on the paternally inherited allele, from which three protein-coding genes (*Dlk1*, *Rtl1* and *Dio3*) are expressed^16^. *Meg3* gene deletion, either by targeting of *Meg3* or IG-DMR, is embryonically lethal and different phenotypes are observed depending on the knock-out (KO) model^17–19^. In addition, *Meg3* seems to act as a tumor suppressor and as an important regulator of cellular proliferation^14,15^. Interestingly, the imprinted gene network was described to be predominantly expressed in hematopoietic stem cells, including Meg3^20^. Moreover, Qian and colleagues recently reported that IG-DMR is essential to maintain fetal liver HSCs^21^. Qian *et al*. performed an in-depth study using a global constitutive KO approach to show that the long-term repopulating capacity of fetal liver HSCs is impaired upon loss of imprinting of the *Dlk1-Meg3* locus. Fetal liver HSCs and adult HSCs greatly differ in their cellular properties such as cycling^22–24^. Thus, due to the specific expression of *Meg3* in adult HSCs, we aimed to address the role of *Meg3* in adult mouse hematopoiesis. Since constitutive *Meg3*-*Dlk1* knockout mouse models are embryonically lethal, we employed a floxed *Meg3* mouse model created by Klibanski and colleagues (Klibanski *et al*. unpublished) to generate inducible HSC *Meg3* knockout mice. Here, we provide genetic evidence that *Meg3* in adult HSCs is dispensable for adult hematopoiesis not only during homeostasis and recovery from inflammatory conditions, but also for reconstitution upon HSC transplantation.

## Results

### Loss of *Meg3* expression does not impair adult hematopoiesis

**Figure 1:**
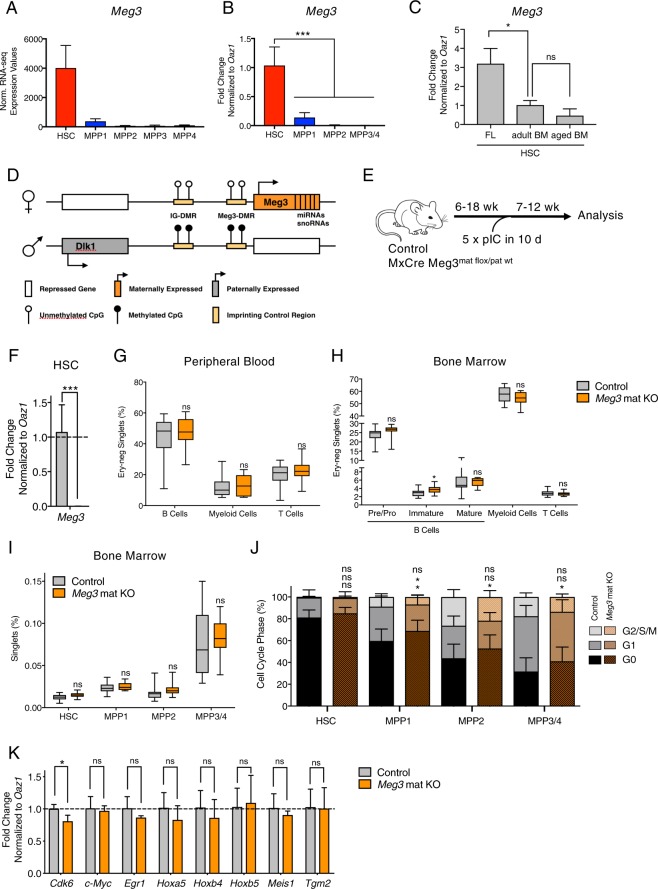
*Meg3* is highly and specifically expressed in HSCs, however *Meg3* mat KO mice exhibit normal hematopoiesis. (**A**,**B**) *Meg3* expression was analyzed by RNA-seq (data derived from^8^) (**A**, n = 3–4, mean + SD) or qPCR analysis (**B**, n = 3, mean + SD, one-way ANOVA) in sorted homeostatic HSCs (LSK CD48− CD150+ CD34− (CD135−)), MPP1 (LSK CD48− CD150+ CD34+ (CD135−)), MPP2 (LSK CD48+ CD150+ (CD135−)), MPP3 (LSK CD48+ CD150− (CD135−)), MPP4 (LSK CD48+ CD150− (CD135+)) or MPP3/4 (LSK CD48+ CD150−) cells. For details on employed surface marker combinations see Supplementary Fig. 1A and Material and Methods (**C**) *Meg3* expression in fetal liver (FL) E13.5, adult bone marrow (BM) and aged BM HSCs analyzed by qPCR. n = 3, mean + SD, student’s t-test. For details on employed surface marker combinations see Supplementary Fig. 1A and Material and Methods (**D**) Schematic Representation of the Dlk1-Meg3 locus (Modified after^38^). (**E**) Workflow of Cre induction by pIC and consecutive analysis. (**F**) qPCR analysis of *Meg3* in sorted control and *Meg3* mat KO HSCs. n = 7, mean + SD, unpaired student’s t-test. (**G**,**H**) Flow cytometry-based analysis of differentiated PB (**G**) and BM cells (**H**). Percent of erythrocyte negative cells is depicted. n = 12–18, unpaired student’s t-test. (**I**) HSC, MPP1, MPP2, and MPP3/4 frequencies determined by flow cytometry analysis. Percentage of BM HSC/MPPs are represented. n = 11–16, unpaired student’s t-test. (**J**) Cell cycle analysis of HSCs/MPPs. Relative percentage within each cell-cycle phase is shown. n = 9–15, mean + SD, two-way ANOVA. (**K**) qPCR analysis of HSC signature genes in sorted control and *Meg3* mat KO HSCs. n = 3, mean + SD, unpaired student’s t-test. For all experiments: *p < 0.05; **p < 0.01; ***p < 0.001, ns: not significant. n indicates number of biological replicates; all panels: 3 independent experiments.

RNA-seq analysis of adult HSC and MPP populations revealed the lncRNA *Meg3* to be highly and specifically expressed in HSCs when compared to progenitors (Fig. 1A)^8^. We confirmed these observations by qPCR analysis (Fig. 1B). *Meg3* expression is high in HSCs independent of age and decreases from the fetal liver towards the aged bone marrow (BM) stage (Fig. 1C). To investigate the functional role of *Meg3* in adult HSCs, we used an inducible transgenic mouse model in which exon 1 to 4 of the *Meg3* allele are floxed (Meg3^mat-flox/pat-flox^, Fig. 1D). We crossed female Meg3^mat-flox/pat-flox^ mice to male MxCre driver mice to generate MxCre Meg3^mat-flox/pat-wt^ mice (from now on *Meg3* mat KO)^25^. The *Meg3* locus is imprinted and *Meg3* is only expressed from the maternally inherited allele harboring unmethylated DMRs (Fig. 1D). To delete *Meg3* in the hematopoietic compartment, we injected adult mice with Poly(I:C) (pIC) to induce Cre expression (Fig. 1E). We kept KO mice for a minimum of 7 weeks prior to analysis to allow recovery of the hematopoietic system to a homeostatic state. After this recovery phase, we sacrificed mice and analyzed primary and secondary hematopoietic organs. First, we confirmed KO efficiency by sorting HSCs (Lineage- Sca1+ Kit+ (LSK) CD150+ CD48− CD34−) and performing qPCR analysis (Fig. 1F, Supplementary Fig. 1A). Deletion of the maternal allele was sufficient to completely disrupt *Meg3* expression. In addition, we analyzed differentially expressed miRNAs by “small RNA-Seq” from LSK CD150+ CD48− cells (Supplementary Fig. 1B). We detected 12 mature miRNAs to be differentially expressed between KO and control cells. Ten of these miRNAs belong to the *Dlk1*-*Meg3* locus and were all found to be strongly downregulated in *Meg3* KO cells. However, we observed no differences in lineage composition in the peripheral blood as determined by flow cytometry analysis. The numbers of B cells, T cells as well as myeloid cells were not affected by loss of *Meg3* expression (Fig. 1G, Supplementary Fig. 1C). Next, we analyzed total blood counts of white blood cells, neutrophils and lymphocytes and observed no significant differences (data not shown). Subsequently, we analyzed the BM composition and in line with peripheral values, we observed no differences in mature cells (myeloid, B, T cells) between control and *Meg3* mat KO mice (Fig. 1H, Supplementary Fig. 1D). Similarly, we did not detect any signs of impairment in spleen and thymus upon *Meg3* deletion (Supplementary Fig. 1E,F). Next, we analyzed the effects of *Meg3* deletion on the HSC/MPP compartment. Our analyses did not reveal any differences in HSC and MPP frequencies (Fig. 1I, Supplementary Fig. 1G). We observed a minor increase in G0 of MPPs in cell cycle analysis, however no differences were observed in HSCs (Fig. 1J, Supplementary Fig. 1H). To analyze the molecular profile of *Meg3* mat KO HSCs, we performed qPCR analysis of HSC-specific and -regulatory genes in control and KO cells (Fig. 1K). Genes were selected based on gene expression signatures identified in previous publications^8,9^. Except for slightly reduced *Cdk6* levels in *Meg3* mat KO HSCs, we did not observe any deregulation of HSC-specific genes upon loss of *Meg3* expression. Collectively, our data show no apparent impairment of adult hematopoiesis in *Meg3* mat KO mice despite the high and specific expression of the lncRNA *Meg3* in adult HSCs.

### *Meg3* expression is dispensable for HSC engraftment and long-term function in serial BM transplantation experiments

**Figure 2:**
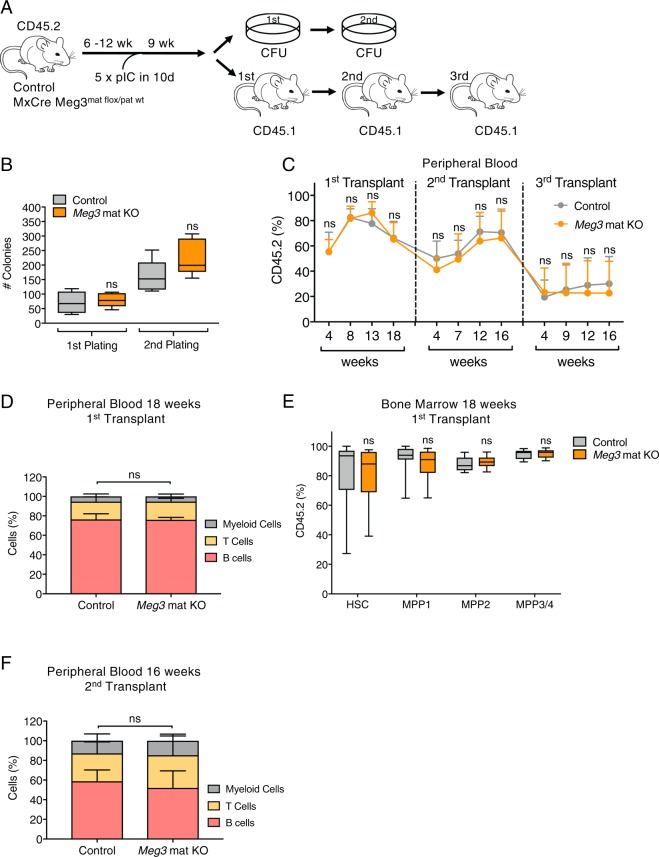
Reconstitution of full chimeras and lineage output is not impaired in *Meg3* mat KO mice. (**A**) Workflow depicting Cre induction and functional analysis by colony forming unit (CFU) assays and generation of full chimeras. (**B**) Functional analysis by serial CFU assays. n = 5–6, unpaired student’s t-test. (**C**) Flow cytometry analysis of serial transplantation experiments. CD45.2% outcome is shown. n = 9, mean + SD, two-way ANOVA. (**D**) Lineage output in PB of primary recipients 18 weeks after transplantation as determined by flow cytometry analysis in CD45.2+ cells. n = 9, mean + SD, two-way ANOVA. (**E**) Flow cytometry analysis of HSC/MPP cells in BM of primary recipients 18 weeks after transplantation. CD45.2% outcome is shown. n = 9, unpaired student’s t-test. (**F**) Lineage output in PB of secondary recipients 16 weeks after transplantation as determined by flow cytometry analysis in CD45.2+ cells. n = 9, mean + SD, two-way ANOVA. For all experiments: *p < 0.05; **p < 0.01; ***p < 0.001, ns: not significant. n indicates number of biological replicates; B: 2 independent experiments; C-F: 1 independent experiment.

Analysis of steady-state hematopoiesis 7 to 12 weeks after MxCre mediated *Meg3* deletion may not necessarily reveal functional HSC impairment as contribution of HSCs to mature cell output during homeostasis is small^26,27^. To functionally evaluate the effects of *Meg3* loss in HSCs, we performed *in vitro* colony forming unit (CFU) assays and *in vivo* serial transplantation assays (Fig. 2A). We did not detect functional defects in adult *Meg3* mat KO HSC/MPPs in serial CFU assays (Fig. 2B). In addition, we analyzed engraftment and lineage output in full chimeras and did not observe differences upon *Meg3* mat KO (Fig. 2C–F). Overall, we detected comparable engraftment of BM cells (CD45.2) in primary lethally irradiated recipients (CD45.1) 4 weeks after transplantation between KO and control cells (Fig. 2C, Supplementary Fig. 2A). Contribution of transplanted cells to the peripheral blood was not altered by *Meg3* mat KO over time in primary recipients. We performed endpoint analysis after 18 weeks and observed no lineage bias of the transplanted cells in the peripheral blood (Fig. 2D). In addition, we conducted analysis of the BM and detected comparable engraftment of *Meg3* mat KO and control HSCs and MPPs (Fig. 2E). To ensure that *Meg3* mat KO was maintained in HSCs and no escapees grew out, we performed qPCR analysis of sorted CD45.2+ HSCs from 18-week primary recipients (Supplementary Fig. 2B). We did not detect *Meg3* expression in KO HSCs, confirming stable KO and contribution of KO cells to lineage output. Furthermore, we did not observe differences in the differentiated lineages in the BM and the spleen (Supplementary Fig. 2C,D).

To investigate long-term engraftment potential, we performed serial transplantation assays. Re-transplantation of total BM cells into secondary recipients did not show impaired engraftment and contribution capacity of *Meg3* mat KO HSCs (Fig. 2C). Analysis of secondary recipients 16 weeks after transplantation revealed no differences in lineage output in the peripheral blood (Fig. 2F). Finally, we performed tertiary transplantation assays and again failed to detect differences between control and KO settings (Fig. 2C). No differences in peripheral blood contribution and generation of B cells, T cells or myeloid cells were detected in serial BM transplantation assays (Supplementary Fig. 2E–G). Overall, we conclude that long-term engraftment and functional potential of adult *Meg3* mat KO HSCs is not impaired indicating that *Meg3* is dispensable for adult hematopoiesis.

### Loss of *Meg3* expression does not impair the competitive potential of HSCs

**Figure 3:**
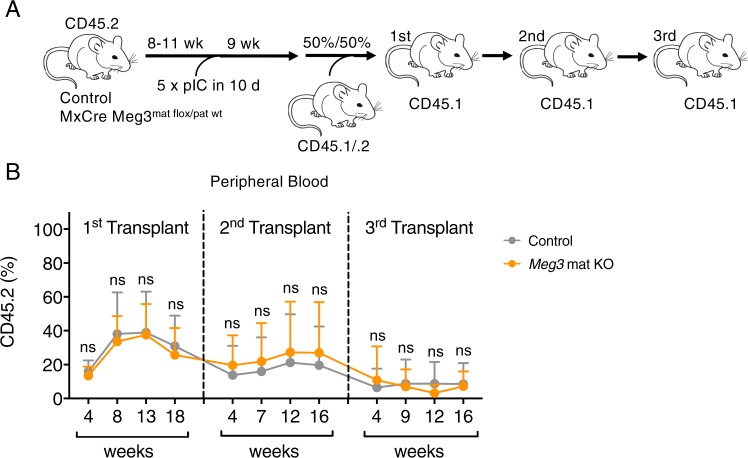
No functional impairment of *Meg3* mat KO BM is detected in serial competitive repopulation experiments. (**A**) Workflow depicting Cre induction and functional analysis by serial competitive transplantation experiments. (**B**) Flow cytometry analysis of serial transplantation experiments. CD45.2% outcome is shown. n = 7–9, mean + SD, two-way ANOVA. n(primary) = 9, n(secondary) = 9, n(tertiary) = 7–9, unpaired student’s t-test. For all experiments: *p < 0.05; **p < 0.01; ***p < 0.001, ns: not significant. n indicates number of biological replicates; 1 independent experiment.

To ensure that *Meg3* mat KO HSCs hold equal functional capacities as wildtype HSCs, we set up 50/50 competitive transplantation experiments. 9 weeks after *Meg3* deletion in donor mice, we mixed total BM cells of CD45.2 *Meg3* mat KO or control mice with CD45.1/2 total BM cells and transplanted them into lethally irradiated CD45.1 recipient mice (Fig. 3A). 4 weeks after transplantation, we did not detect competitive disadvantages in transplanted *Meg3* mat KO cells compared to control cells (Fig. 3B, Supplementary Fig. 3A).

As for full chimeras, we performed serial transplantation assays by re-transplanting total BM cells into secondary and tertiary recipients, respectively. We did not observe any competitive disadvantages in peripheral blood contribution over time (Fig. 3B). In addition, no differences in peripheral blood contribution and generation of B cells, T cells or myeloid cells were detected (Supplementary Fig. 3B–D). These findings show that even in competitive transplantation settings, HSCs lacking *Meg3* expression do no exhibit functional impairments or deficiencies.

### The lncRNA *Meg3* is dispensable in HSCs expressing *Vav1*

**Figure 4:**
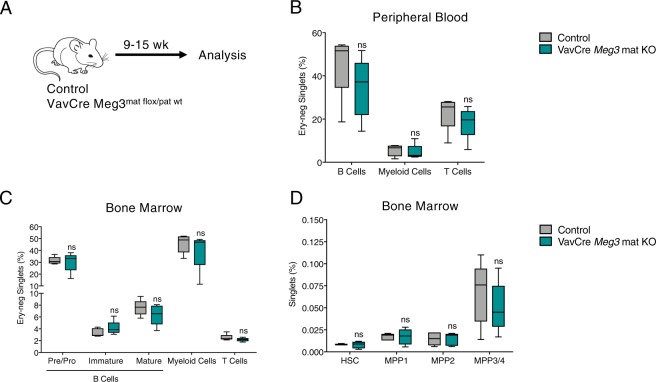
VavCre *Meg3*^mat-flox/pat-wt^ mice are viable and exhibit normal hematopoiesis. (**A**) Workflow. (**B**,**C**) Flow cytometry-based analysis of differentiated PB (**B**) and BM cells (**C**). Percent of erythrocyte negative cells are depicted. n = 5, unpaired student’s t-test. (**D**) HSCs, MPP1, MPP2, and MPP3/4 frequencies determined by flow cytometry analysis. Percentage of BM HSC/MPPs are represented. n = 5, unpaired student’s t-test. For all experiments: *p < 0.05; **p < 0.01; ***p < 0.001, ns: not significant. n indicates number of biological replicates; B-D: 2 independent experiments.

Constitutive loss of imprinting of the *Dlk1-Meg3* locus leads to impairments in fetal liver HSCs^21^. To understand the role of *Meg3* in definitive HSCs during embryogenesis we generated VavCre *Meg3*^mat-flox/pat-wt^ mice. VavCre mediated deletion has been detected in embryonic stage 11.5 CD45+ aorta–gonad–mesonephro (AGM) and CD45+ fetal liver cells^28^. Thus, *Meg3* is deleted in all cells expressing *Vav1*, leading to deletion of *Meg3* after the endothelial-cell-to-HSC transition during embryogenesis. VavCre *Meg3*^mat-flox/pat-wt^ mice were born and appeared healthy (data not shown). In depth analysis of the hematopoietic system was performed 9–15 weeks after birth (Fig. 4A). Analysis of differentiated cells in the peripheral blood and in the bone marrow did not reveal any alterations in adult mice upon loss of *Meg3* in HSCs during embryonic development (Fig. 4B,C). In addition, we analyzed the HSC/MPP compartment and did not observe any changes compared to control mice (Fig. 4D). Complete loss of *Meg3* expression was confirmed in adult HSCs by qPCR analysis (data not shown). In summary, *Meg3* is not only dispensable in adult HSCs but hematopoiesis is also not impaired upon loss of *Meg3* expression in *Vav1*-expressing hematopoietic cells during embryogenesis.

### Interferon-mediated HSC stress response is marginally affected by lack of *Meg3* expression

**Figure 5:**
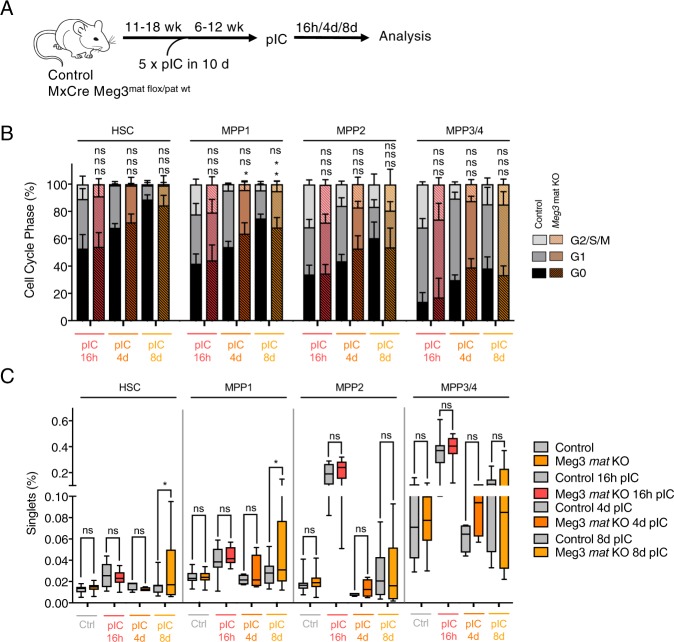
HSC stress response is only marginally affected in *Meg3* mat KO HSCs. (**A**) Workflow depicting pIC-mediated Cre and interferon-mediated stress induction. (**B**) Flow cytometry-based cell cycle analysis of HSCs/MPPs. Relative percentage within each cell-cycle phase is shown. n = 4–11, mean + SD, two-way ANOVA. (**C**) HSCs, MPP1, MPP2, and MPP3/4 frequencies determined by flow cytometry analysis. Percentage of BM HSC-MPPs are represented. n = 4–18, one-way ANOVA. For all experiments: *p < 0.05; **p < 0.01; ***p < 0.001, ns: not significant. n indicates number of biological replicates; B-C: 1–4 independent experiments.

As *Meg3* is known to be involved in cell cycle control, we asked whether *Meg3* deficiency could impair HSC activation or return to quiescence in response to inflammatory stress conditions. To investigate the interferon-mediated response in HSCs, we performed additional pIC injections 6 to 12 weeks following *Meg3* deletion (Fig. 5A). pIC is a dsRNA-analogue and induces a type I interferon (IFNα/β) response, which strongly induces proliferation in the HSC/MPP compartment, including exit from G_0_^5^. Cells become activated within 16 h after injection and return to quiescence within 8 days. We analyzed the effects of pIC treatment 16 h, 4 days and 8 days after injection to monitor the stress response (Fig. 5A). As expected, HSCs and MPP cells exit G_0_ 16 h after pIC injection (Fig. 5B). This proliferative response was not impaired in any of the analyzed HSC/MPP subsets upon loss of *Meg3* expression. From 4 to 8 days we observed a stepwise return to the respective cell cycle status of HSCs and MPPs. We did not see any significant differences between control and *Meg3* mat KO HSCs, MPP2 and MPP3/4 cells in the return to a quiescent state. MPP1 cells lacking *Meg3*, however, showed a slightly increased percentage of cells in G_0_ after 4 days and a slightly decreased percentage of cells in G_0_ after 8 days compared to the respective control, indicating a marginally decreased return of mutant cells to quiescence.

In addition to the cell cycle state, we monitored the frequency of the HSC/MPP compartments in the BM (Fig. 5C). As expected, we detected an increase in the respective compartments 16 h after pIC injection that can be explained by the proliferative response^5^. Over time (4–8 days), the frequency of the respective HSC/MPP compartment was reduced again. This response was not impaired in *Meg3* mat KO HSCs and MPPs. Only in HSC and MPP1 cells we observed slightly increased frequencies 8 days after pIC injection in *Meg3* mat KO animals. To address whether this slight increase was of biological relevance, we performed serial colony forming unit assays 16 h and 8 days after pIC injection (Supplementary Fig. 4A). We did not observe any significant differences in colony forming capacity in serial plating between control and *Meg3* mat KO cells, even though we observed a slight increase in HSC frequency upon *Meg3* mat KO during recovery from stress response (Fig. 5C). We conclude, that the slight increase in the HSC compartment during stress recovery is not of major biological relevance for the hematopoietic system. To further strengthen our observations, we performed serial stress experiments (Supplementary Fig. 4B). Ten weeks after *Meg3* deletion, mice were injected with pIC for 4 weeks. After an 8-week recovery phase, mice were sacrificed and analyzed. As expected, serial stress led to a reduction in HSC/MPP frequencies. This effect was not altered in *Meg3* mat KO cells. Overall, this indicates that *Meg3* is not required for the proliferative response and recovery upon interferon-mediated inflammatory stress.

## Discussion

*Meg3* is known to play an important functional role in embryonic development as shown in several mouse genetic studies^17–19^. The severity of the observed developmental phenotypes depends on the applied genetic targeting strategy. For example, IG-DMR KO and *Meg3* KO models were recently used to investigate the effects of *Dlk1*-*Meg3* imprinting loss on fetal liver HSCs^17,18,21^. Using these models, Qian *et al*. observed reduced fetal liver HSC frequency and showed their functional impairment by transplantation into adult mice. Interestingly, expression patterns of the *Dlk1-Meg3* locus in fetal liver and adult HSCs seem to be highly conserved^21^. Here we employed an inducible MxCre Meg3^mat-flox/pat-wt^ mouse model to induce deletion of *Meg3* in mice after the establishment of adult homeostatic hematopoiesis. Thereby, we were able to directly investigate the consequences of loss of *Meg3* expression on adult hematopoiesis, excluding potential developmental and microenvironmental impairments that could eventually affect HSCs. Since adult HSCs are deeply quiescent/dormant and *Meg3* is known to be involved in cell cycle regulation via the p53- and Rb-pathways, we used straight and competitive transplantation assays to challenge HSCs and to further investigate HSC function over time^22,29–31^. We performed extensive analyses and observed no biologically relevant differences in homeostatic and stressed hematopoiesis upon loss of *Meg3* expression. To address the role of *Meg3* during embryonic development, we deleted *Meg3* in CD45+ cells in the murine embryo by using *Vav1* as a Cre driver. Surprisingly, mice were viable and no signs of hematopoietic impairments were observed in adult mice. Therefore, *Meg3* seems to be dispensable for embryonic establishment of hematopoiesis and *Meg3* does not seem to be important for HSC function after the endothelial-cell-to-HSC transition^28^. However, *Meg3* might be important for this transition step during development leading to the defects in fetal liver HSCs observed by Qian *et al*.^21^. Importantly, our model is targeting exon 1–4 of the *Meg3* locus, leading to loss of *Meg3* expression and loss of expression of the miRNA cluster, and does not target DMRs. A broader disruption of the imprinted locus could potentially lead to globally altered expression patterns, which in turn could affect hematopoiesis – independent of *Meg3* and the miRNA cluster itself. Future studies will need to shed light on the effects of DMR targeting affecting the entire *Meg3-Dlk1* locus in adult hematopoiesis. *Meg3* is additionally known to act as a tumor suppressor in several types of cancer including leukemia^14,15,32^. *Meg3* has been shown to inhibit tumor cell proliferation *in vitro* and *in vivo* and acts via a p53-dependent and -independent axis in acute myeloid leukemia cell lines^15,32^. In our study, we show that loss of *Meg3* alone did not lead to the development of leukemia or myeloproliferative diseases even in long-term experiments such as tertiary transplantations. Furthermore, no proliferative advantages of *Meg3* mat KO cells were observed. Thus, based on these observations the loss of the tumor suppressor *Meg3* alone is not sufficient for cell transformation in the hematopoietic system. In line with our results, loss of *Meg3* mostly occurs upon epigenetic silencing supporting the concept that *Meg3* dysregulation might result as a secondary event during the development of leukemia^15^. Future studies will be necessary to better understand the role of *Meg3* in development and maintenance of leukemia.

## Methods

### Mice

*Meg3* flox mice were generated by Klibanski *et al*., loxP sites flank exon 1 to 4 (Klibanski, unpublished). Female *Meg3* flox mice were crossed to male MxCre driver mice^25^, generating MxCre *Meg3*^mat-flox/pat-wt^ mice. Cre+ animals were used in experiments. As control mice, MxCre mice or *Meg3*^mat-flox/pat-wt^ mice were used. All animals were injected 5 times over 10 days intraperitoneally with pIC (100 µg pIC in PBS) 6–18 weeks after birth. Analysis or further experiments were started 6–12 weeks after deletion. Female *Meg3* flox mice were crossed to male VavCre driver mice^33^ for analysis of embryonic *Meg3* deletion, generating VavCre *Meg3*^mat-flox/pat-wt^ mice. Mice were analyzed 9–15 weeks after birth. As control mice, *Meg3*^mat-flox/pat-wt^ littermates were used. C57BL/6 J mice, 11–17 weeks old (CD45.2, CD45.1 or CD45.2/CD45.1) were either purchased from Harlan Laboratories (now Envigo) (the Netherlands) or Janvier Labs (France) or bred in-house. Euthanization of mice by cervical dislocation and all animal procedures were performed according to protocols approved by the German authorities, Regierungspräsidium Karlsruhe (Nr. A23/17, Z110/02, DKFZ 299 and G149-16). Generation of “small RNA-Seq data” was performed using residual material from *Meg3* KO and control mice generated during the reported experiments under the test number G149-16

### Cell Suspension and Flow Cytometry

#### Peripheral Blood and BM Analysis

For isolation of BM, spleen and thymus cells, mice were euthanized by cervical dislocation. Femur, tibia, hip and spine bones, spleen and thymus were isolated. Bones were crushed in ice-cold PBS using pistil and mortar. The resulting cell suspension was filtered through a 40 µM cell strainer and pelleted by centrifugation. Total BM cells were used for colony forming unit or transplantation assays. For flow cytometry analysis and FACS, erythrocytes were lysed using ACK Lysing Buffer (Lonza, Basel, Switzerland). BM cells were counted using a Vi-CellXR Cell Viability Analyzer (Beckman Coulter, Brea, CA, USA) and used for flow cytometry stainings.

#### FACS

For FACS, total BM cells were lineage depleted by using the Dynabeads Untouched Mouse CD4 Cells Kit (Invitrogen, Carlsbad, CA, USA). Briefly, total BM was stained with a 1:5 dilution of the Lineage Cocktail provided in the Kit for 30 min. Cells were incubated for 20 min with 1.5 mL beads per mouse of washed Dynabeads. A magnet was used for cell depletion to enrich for the lineage-negative cell fraction. To purify HSCs, the depleted cells were stained for 30 min using the following monoclonal antibodies: anti-lineage [anti-CD4 (clone GK1.5), anti-CD8a (53-6.7), anti-CD11b (M1/70), anti- B220 (RA3-6B2), anti-GR1 (RB6-8C5) and anti-TER119 (Ter-119)] all PE-Cy7; anti-CD117/c-Kit (2B8)-PE; anti-Ly6a/Sca-1 (D7)-APC-Cy7; anti-CD34 (RAM34)-FITC; anti-CD150 (TC15-12F12.2)-PE-Cy5; anti-CD48 (HM48-1)-PB. For isolation of transplanted HSCs, the following monoclonal antibodies were used: anti-lineage [anti-CD4 (clone GK1.5), anti-CD8a (53-6.7), anti-CD11b (M1/70), anti- B220 (RA3-6B2), anti-GR1 (RB6-8C5) and anti-TER119 (Ter-119)] all AF700; anti-CD117/c-Kit (2B8)-APC; anti-Ly6a/Sca-1 (D7)-APC-Cy7; anti-CD34 (RAM34)-FITC; anti-CD150 (TC15-12F12.2)-PE-Cy5; anti-CD48 (HM48-1)-PE; anti-CD45.1 (A28)-PE-Cy7; anti-CD45.2 (104)-PB. Monoclonal antibody conjugates were purchased from eBioscience (Thermo Fisher, Waltham, MA, USA) or BioLegend (San Diego, CA, USA). Cell sorting was performed on a FACS Aria II (Becton Dickinson). Sorted cells were collected into RNA lysis buffer (ARCTURUS PicoPure RNA Isolation Kit (Life Technologies, Invitrogen, Carlsbad, CA, USA)) for qPCR analysis and stored at −80 °C.

#### Flow Cytometry

Ery-lysed peripheral blood and BM cells were used for flow cytometry stainings. Supplementary Table 1 defines the monoclonal antibodies applied for the different surface staining approaches. Cells were stained for 30 min at 4 °C. For cell cycle stainings, surface stainings were performed and cells were fixed and permeabilized by using BD Cytofix/Cytoperm Buffer (Beckton Dickinson, Franklin Lakes, NJ, USA). Subsequently, intracellular Ki-67 (B56)-AF647 (BD Biosciences, Franklin Lakes, NJ, USA) staining was performed in PermWash solution (Beckton Dickinson, Franklin Lakes, NJ, USA). Then cells were stained with Hoechst 33342 (Invitrogen, Carlsbad, CA, USA) for 15 min at RT. Monoclonal antibody conjugates were purchased from eBioscience (Thermo Fisher, Waltham, MA, USA) or BioLegend (San Diego, CA, USA).

### Gene Expression Analysis by qPCR

HSCs (MxCre or MxCre Meg3^mat flox/pat wt^ LSK CD150+ CD48− CD34− or fetal liver HSCs: embryonic stage 13.5 CD48− CD41+ cKit+ CD34+; adult HSCs (8–12 weeks): LSK CD150+ CD48− CD34− CD135−; aged HSCs (24 month): LSK CD150+ CD48− CD34− CD135−) were FACS sorted into RNA lysis buffer (ARCTURUS PicoPure RNA Isolation Kit (Life Technologies, Invitrogen, Carlsbad, CA, USA)) for qPCR analysis and stored at −80 °C. RNA was isolated using the ARCTURUS PicoPure RNA Isolation Kit (Life Technologies, Invitrogen, Carlsbad, CA, USA) following manufacturer’s instructions. For cDNA synthesis, reverse transcription was performed using the SuperScript VILO cDNA Synthesis Kit (Invitrogen, Carlsbad, CA, USA) according to manufacturer’s guidelines. Fast SYBR Green Master Mix (Thermo Fisher, Waltham, MA, USA) was used on a ViiA 7 Real-Time PCR System (Applied Biosystems, Foster City, CA, USA) for qPCR analysis. RNA expression was normalized to the housekeeping gene *Oaz1* and presented as relative quantification (Ratio = 2^−DDCT^). For Primer Sequences see Supplementary Table 2.

### Small RNA Sequencing

#### Sample Preparation and Library Generation

Approximately 10,000–20,000 LSK SLAM cells (LSK CD150+ CD48−) were FACS sorted into 100 µl QIAzol Lysis Reagent (Qiagen, Venlo, Netherlands). Total RNA Extraction was performed by adding 20 µl Chloroform (Sigma-Aldrich, St. Louis, MO, USA). Samples were incubated at RT and centrifuged at 12000 rpm for 5 min at RT. The aqueous phase was taken and 0.4 µl glycoblue (Thermo Fisher, Waltham, MA, USA) and 75 µl Isopropanol were added per sample. Samples were stored at −20 °C for at least 5 days. Samples were centrifuged at 13000 rpm for 1 h at 4 °C. Samples were washed with 70% Ethanol and centrifuged at 13000 rpm for 15 min at 4 °C. Supernatant was discarded and the pellet was dried for 1 min. The Pellet was resuspended in 8 µl H2O. RNA quality was analyzed using the Agilent RNA 6000 Pico Kit (Agilent Technologies, Santa Clara, CA, USA) according to manufacturer’s instructions. Libraries for small RNA-Seq were generated using the SMARTer® smRNA-Seq Kit for Illumina® (Takara Bio Inc., Kusatsu, Shiga Prefecture, Japan) according to manufacturer’s instructions. 23 ng of total RNA were used and 15 cycles were applied for PCR amplification. Quality was checked using the Agilent High Sensitivity DNA Kit (Agilent Technologies, Santa Clara, CA, USA) according to manufacturer’s instructions. Size selection was performed by using AMPure XP Beads (Beckman Coulter, Brea, CA, USA) according to manufacturer’s instructions. Sequencing was performed on a HiSeq 2000 device (Illumina, San Diego, CA, USA).

### MiRNA Sequencing Analysis

#### Genomic Mapping

The adapter sequences ATGATCGGAAGAG, GATGAAGAACGCAG, AGATCGGAAGAGCGTCGTGTAGGGAAAGAGTGT, GGTCTACG, TGGAATTCTCGGGTGCCAAGG, TCGTATGCCGTCTTCTGCTTG, AGATCGGAAGAGCACACGTCTGAACTCCAGTCA were removed with Trimmomatic^34^ (version 0.32, parameters: SE ILLUMINACLIP:adapter:2:30:10 HEADCROP:4 TRAILING:3 MINLEN:17) from the fastq sequences, additionally all reads containing N and all sequences <17 bases were removed. HomerTools^35^ (v3.18, trim -3 AAAAAAAAA) for removing polyA tails and fastx_artifacts_filter (FASTX Toolkit 0.0.13 http://hannonlab.cshl.edu/fastx_toolkit/) were used for further cleaning. Then the clean fastq files were mapped against mouse genome 38 (NCBI) using Bowtie 2 version 2.2.4^36^ (parameters -N 0 -L 8). The resulting SAM files were converted to BAM and sorted using samtools version 1.3.1.

#### miRNA Mapping and Counting

The clean fastq files were mapped with bowtie version 0.12.9 (-f -v 1 -a–best -S) against an assembled ncRNA database consisting of a unique set of miRNAs of Ensembl version 95 (http://www.ensembl.org/) and miRBase (v21, http://www.mirbase.org/). The resulting SAM files were parsed and the number of reads for each mature miRNA was counted using Perl.

#### Comparison of Control Cre and KO samples

For the comparison with DESeq. 2^37^ the input tables containing the replicates for the groups to compare were created by a custom perl script. In the count matrix rows with an average count number <5 were removed, then DESeq. 2 (version 1.4.1) was run with default parameters.

### Transplantation Experiments

#### Full Chimeras

1 × 10^6^ total BM cells from induced MxCre control mice or induced MxCre Meg3^mat-flox/pat-wt^ mice were transplanted into fully irradiated (2 × 5 Gy.) CD45.1 mice within 24 h after irradiation by tail vein injection. 18 weeks after transplantation, mice were euthanized and 3 × 10^6^ total BM cells were transplanted into fully irradiated (2 × 5 Gy.) CD45.1 secondary recipient mice. 16 weeks after transplantation, mice were euthanized and 3 × 10^6^ total BM cells were transplanted into fully irradiated (2 × 5 Gy.) CD45.1 tertiary recipient mice. Contribution of CD45.2-donor cells was monitored in peripheral blood approximately every 4 weeks by flow cytometry using the following monoclonal antibodies: anti-CD45.1 (A20)-PECy7, anti-CD45.2 (104)-PB, anti-CD11b (M1/70)-AF700, anti-GR1 (RB6.8C5)-APC, anti-CD8a (53.6.7)-PE, anti-CD4 (GK1.5)-PE, anti-B220 (RA3.6B2)-PE-Cy5.

#### 50/50 Chimeras

2 × 10^5^ total BM cells from induced MxCre control mice or induced MxCre Meg3^mat-flox/pat-wt^ mice were transplanted into fully irradiated (2 × 5 Gy.) CD45.1 mice within 24 h after irradiation by tail vein injection in competition to 2 × 10^5^ total BM cells from CD45.1/2 mice. 18 weeks after transplantation, mice were euthanized and 3 × 10^6^ total BM cells were transplanted into fully irradiated (2 × 5 Gy.) CD45.1 secondary recipient mice. 16 weeks after transplantation, mice were euthanized and 3 × 10^6^ total BM cells were transplanted into fully irradiated (2 × 5 Gy.) CD45.1 tertiary recipient mice. Contribution of CD45.2-donor cells was monitored in peripheral blood approximately every 4 weeks by flow cytometry using the following monoclonal antibodies: anti-CD45.1 (A20)-PECy7, anti-CD45.2 (104)-PB, anti-CD11b (M1/70)-AF700, anti-GR1 (RB6.8C5)-APC, anti-CD8a (53.6.7)-PE, anti-CD4 (GK1.5)-PE, anti-B220 (RA3.6B2)-PE-Cy5.

### pIC Stress Experiments

Mice were injected intraperitoneally with 100 µg pIC (in PBS) or PBS either 8 days, 4 days or 16 h prior to endpoint analysis. For serial stress experiments, mice were injected for a period of 4 weeks 2 times a week. Mice were analyzed 8 weeks after the last pIC injection.

### Statistical Analysis

Statistical analysis was performed by unpaired Student’s t test or two- or one-way ANOVA without correction for multiple comparison (Fisher LSD test). Significance levels were set at *p < 0.05, **p < 0.01 and ***p < 0.001. GraphPad Prism was used for statistical analysis.

## Supplementary information

Supplementary Figures

## Data Availability

The small RNA-Seq dataset generated and analyzed during the current study is available in the ArrayExpress repository, ArrayExpress accession E-MTAB-7519 (http://www.ebi.ac.uk/arrayexpress/).
