# Supplementary material for: The long non-coding RNA Meg3 is dispensable for hematopoietic stem cells
Source: Sci Rep. 2019 Feb 14;9:2110. doi: 10.1038/s41598-019-38605-8 (PMC6375991; doi:10.1038/s41598-019-38605-8)
Supplement: Supplementary file 1 — Supplementary Figures [file 41598_2019_38605_MOESM1_ESM.pdf]

The long non-coding RNA Meg3 is dispensable for hematopoietic stem cells in mice

Pia Sommerkamp[1,2,3], Simon Renders[1,2], Luisa Ladel[1,2], Agnes Hotz-Wagenblatt[4], Katharina Schönberger[1,2,5], Petra Zeisberger[1,2], Adriana Przybylla[1,2], Markus Sohn[1,2], Yunli Zhou[6], Anne Klibanski[6], Nina Cabezas-Wallscheid[1,2,5,*] and Andreas Trumpp[1,2,*]

[1] Division of Stem Cells and Cancer, German Cancer Research Center (DKFZ), 69120 Heidelberg, Germany

[2] Heidelberg Institute for Stem Cell Technology and Experimental Medicine (HI-STEM gGmbH), 69120 Heidelberg, Germany

[3] Faculty of Biosciences, Heidelberg University, 69117 Heidelberg, Germany

[4] Core Facility Omics IT and Data Management, German Cancer Research Center (DKFZ), 69120 Heidelberg, Germany

[5] Max Planck Institute of Immunobiology and Epigenetics, 79108 Freiburg, Germany

[6] Neuroendocrine Unit, Massachusetts General Hospital and Harvard Medical School, Boston, MA 02114, USA

[*] Co-senior author

Correspondence to

Dr. Nina Cabezas-Wallscheid

Stübeweg 51

D-79108 Freiburg

Tel: +49 761 5108-530

E-Mail: cabezas@ie-freiburg.mpg.de

Prof. Dr. Andreas Trumpp

Im Neuenheimer Feld 280

D-69120 Heidelberg

Tel: +49 6221 42 3901

E-Mail: a.trumpp@dkfz.de

Supplementary Information

Supplementary Table 1: Flow cytometry stainings

Staining	Surface Marker	Monoclonal Antibody
Differentiated Cells (Peripheral Blood, BM, Spleen)	CD4	GK1.5 (PE-Cy7)
	CD8a	53-6.7 (PE-Cy7)
	CD11b	M1/70 (APC-Cy7)
	Gr1	RB6-8C5 (APC)
	Ter119	Ter-119 (PB)
	CD71	R17217 (PE)
	B220	RA3-6B2 (AF700)
	IgM	II/41 (PE-Cy5)
HSCs (BM)	Lineage (CD4, CD8a, CD11b, Gr1, Ter119, B220)	Clones see differentiated cells, all PE-Cy7
	CD117	2B8 (BV711)
	Sca-1	D7 (APC-Cy7)
	CD48	HM48-1 (PB)
	CD150	TC15-12F12.2 (PE-Cy5)
	CD34	RAM34 (FITC)
	CD16/32	93 (APC)
	CD127	A7R34 (PE)
Thymus	CD4	GK1.5 (PE-Cy7)
	CD8a	53-6.7 (PB)
	CD25	PC61 (APC)
	CD44	IM7 (PE-Cy5)
Cell Cycle (BM)	Lineage (CD4, CD8a, CD11b, Gr1, Ter119, B220)	Clones see differentiated cells, all AF700
	CD117	2B8 (PE)
	Sca-1	D7 (APC-Cy7)
	CD48	HM48-1 (PE-Cy7)
	CD150	TC15-12F12.2 (PE-Cy5)
	CD34	RAM34 (FITC)
Differentiated Cells Transplants (Peripheral Blood, BM, Spleen)	CD4	GK1.5 (PE-Cy5)
	CD8a	53-6.7 (PE-Cy5)
	CD11b	M1/70 (APC-Cy7)
	Gr1	RB6-8C5 (APC)
	Ter119	Ter-119 (FITC)
	CD71	R17217 (PE)
	B220	RA3-6B2 (AF700)
	CD45.1	A28 (PE-Cy7)
	CD45.2	104 (PB)
HSCs Transplants (BM)	Lineage (CD4, CD8a, CD11b, Gr1, Ter119, B220)	Clones see differentiated cells, all AF700
	CD117	2B8 (BV711)
	Sca-1	D7 (APC-Cy7)
	CD48	HM48-1 (PE)
	CD150	TC15-12F12.2 (PE-Cy5)
	CD34	RAM34 (FITC)
	CD45.1	A28 (PE-Cy7)
	CD45.2	104 (PB)

Supplementary Table 2: Primer Sequences

Primer	Sequence
Cdk6 fwd	cgagtgcagaccagtgagg
Cdk6 rev	tgtgcacacatcaaacaacct
c-Myc fwd	caccagcagcgactctga
c-Myc rev	ggggtttgcctcttctcc
Egr1 fwd	agttccaacgccctctgac
Egr1 rev	acaggaacccgaaccaca
Hoxa5 fwd	agctgcacattagtcacgaca
Hoxa5 rev	gcggttgaagtggaattctt
Hoxb4 fwd	ctggatgcgcaaagttcac
Hoxb4 rev	gtgaaactccttctccaactcc
Hoxb5 fwd	gcttcacatcagccacgata
Hoxb5 rev	caggtagcgattgaagtggaat
Meis1 fwd	gacgctttaaagagagataaagatgc
Meis1 rev	catttctcaaaaatcagtgctaaga
Meg3 fwd	cgaggacttcacgcacaa
Meg3 rev	attccagatgatggctttg
Oaz1 fwd	tttcagctagcatcctgtactcc
Oaz1 rev	gaccctggtcttgtcgttaga
Tgm2 fwd	cctgaccctggatccctact
Tgm2 rev	cacccgctgtacttctcgtag

Sommerkamp et al.

A

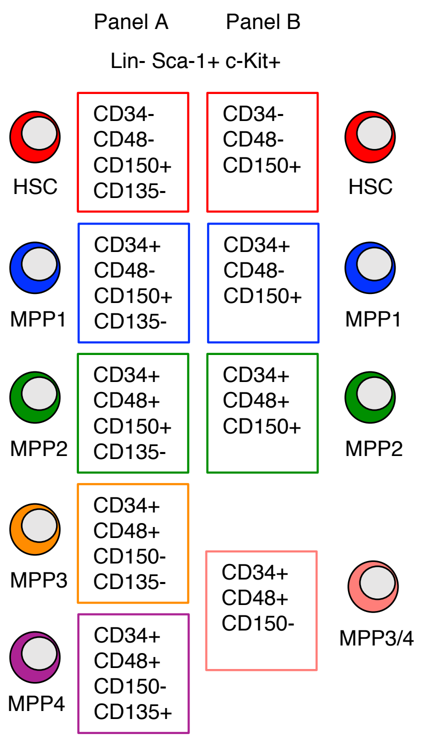

B

Symbol	log2FC	p-value adjusted	Direction in *Meg3* KO	Chr	*Dlk1-Meg3* Locus
mmu-miR-379-5p	-6,3902	0,0038	down	12	Yes
mmu-miR-300-3p	-6,102	0,0311	down	12	Yes
mmu-miR-541-5p	-5,9404	0,0311	down	12	Yes
mmu-miR-376b-5p	-5,9643	0,0311	down	12	Yes
mmu-miR-326-3p	-2,5344	0,0311	down	7	No
mmu-miR-136-3p	-5,3738	0,0375	down	12	Yes
mmu-miR-382-5p	-5,2985	0,0389	down	12	Yes
mmu-miR-376c-5p	-5,6075	0,0553	down	12	Yes
mmu-miR-411-5p	-5,7243	0,0553	down	12	Yes
mmu-miR-134-5p	-5,7421	0,0553	down	12	Yes
mmu-miR-712-5p	-3,2345	0,0553	down	unknown	unknown
mmu-miR-136-5p	-5,6663	0,0588	down	12	Yes

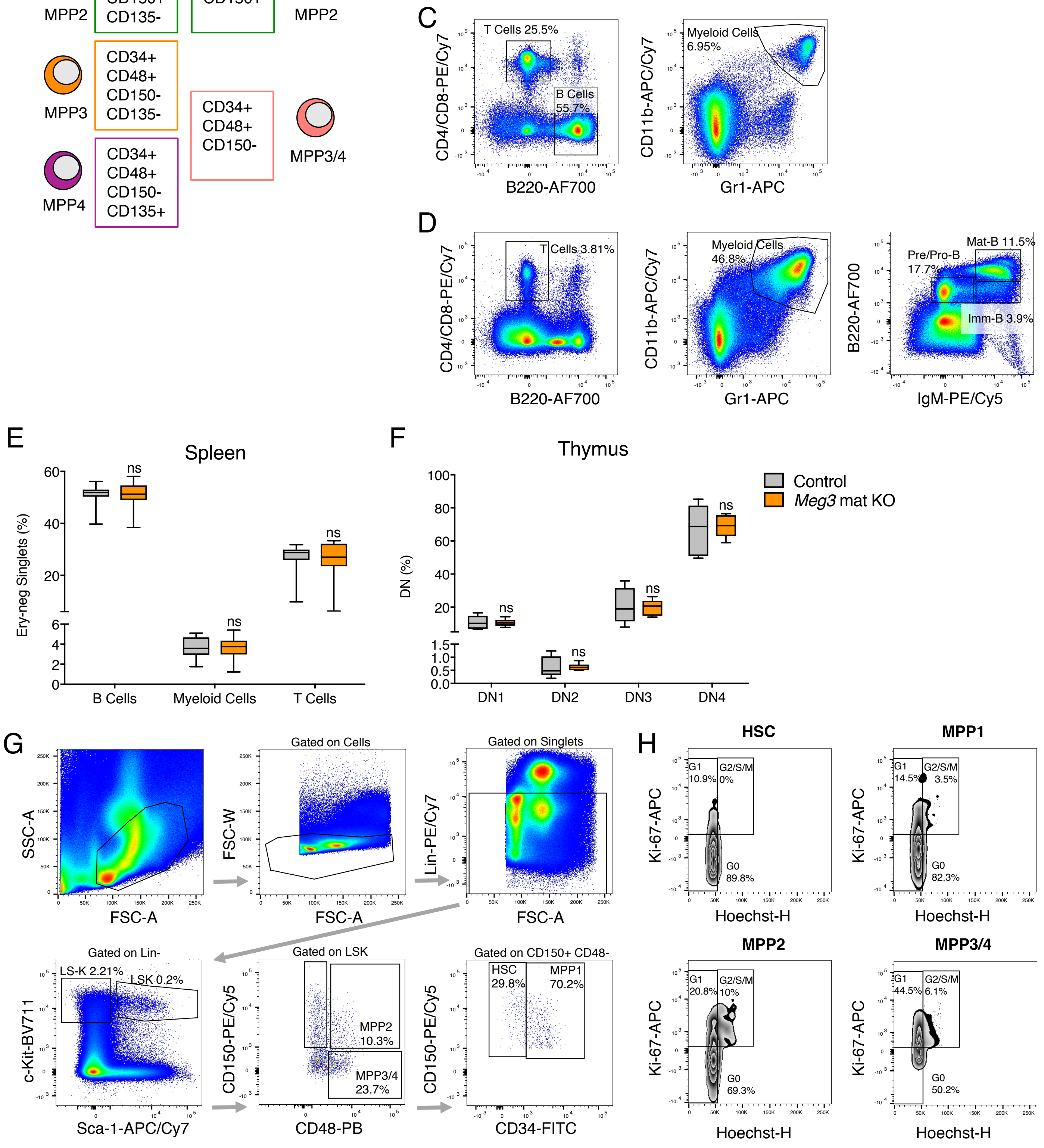

Supplementary Figure 1

Supplementary Figure 1: (**A**) Surface marker profiles of HSCs and MPPs as used in flow cytometry analysis and sorting of BM cells. CD135 was only included for analysis in Fig. 1 A and 1 C (Panel A), for all other experiments Panel B was used. (**B**) Differential mature miRNA expression detected by small RNA-Seq in sorted LSK CD150+ CD48- cells (LSK Slam). Adjusted p-value <0.1 is shown. (**C**) Representative flow cytometry dot plots showing PB analysis. (**D**) Representative flow cytometry dot plots showing analysis of BM differentiated cells. (**E-F**) Flow cytometry analysis of differentiated Spleen (**E**) and double negative (DN) Thymus cells (**F**). n=9-18, unpaired student's t-test. ns: not significant. (**G**) Representative flow cytometry dot plots showing analysis of BM HSC/MPP populations. (**H**) Representative flow cytometry zebra plots (including outliers) showing cell cycle analysis of BM HSC/MPP populations. For all experiments: *p <0.05; **p <0.01; ***p <0.001., ns: not significant. n indicates number of biological replicates; E-F,H: 3 independent experiments.

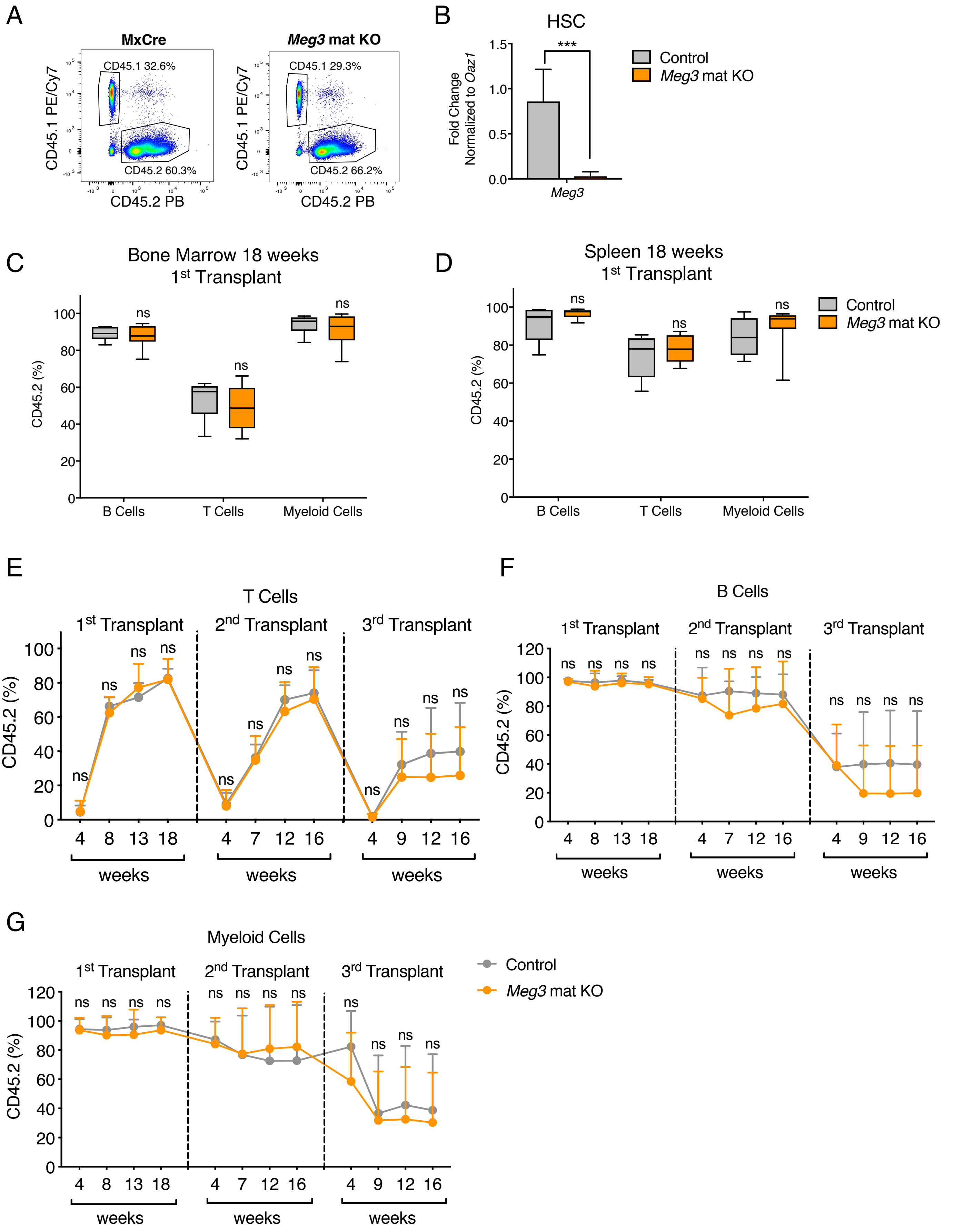

Supplementary Figure 2

Supplementary Figure 2: (**A**) Representative flow cytometry dot plots showing PB analysis of full chimeras. (**B**) qPCR analysis of *Meg3* in sorted CD45.2+ HSCs of primary recipients 18 weeks after transplantation. n=5-6, mean +SD, unpaired student's t-test. (**C-D**) Flow cytometry analysis of differentiated BM (**C**) and Spleen cells (**D**) in primary recipients. n=8-9, unpaired student's t-test. (**E-G**) Flow cytometry analysis of serial transplantation experiments. The data for CD45.2% positive cells in the chimeras is shown for T cells (**E**), B cells (**F**) and myeloid cells (**G**). n=7-9, mean +SD, two-way ANOVA. For all experiments: *p <0.05; **p <0.01; ***p <0.001., ns: not significant. n indicates number of biological replicates; B: 3 independent experiments, C-G: 1 independent experiment.

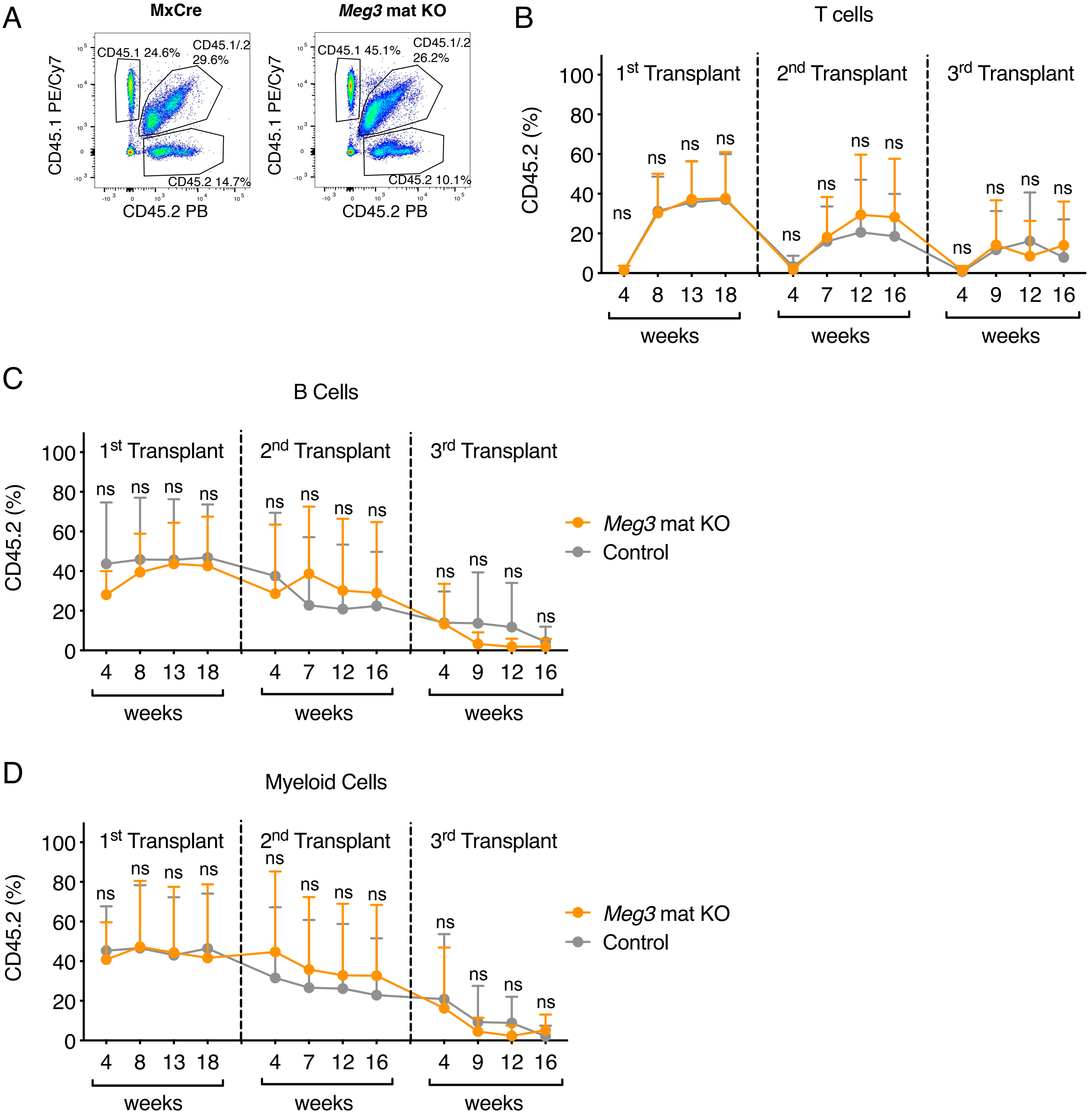

Supplementary Figure 3: (**A**) Representative flow cytometry dot plots showing PB analysis of competitive chimeras. (**B-D**) Flow cytometry analysis of serial transplantation experiments. The data for CD45.2% positive cells in the chimeras is shown for T cells (**B**), B cells (**C**) and myeloid cells (**D**). n=7-9, mean +SD, two-way ANOVA. For all experiments: *p <0.05; **p <0.01; ***p <0.001., ns: not significant. n indicates number of biological replicates; B-D: 1 independent experiment.

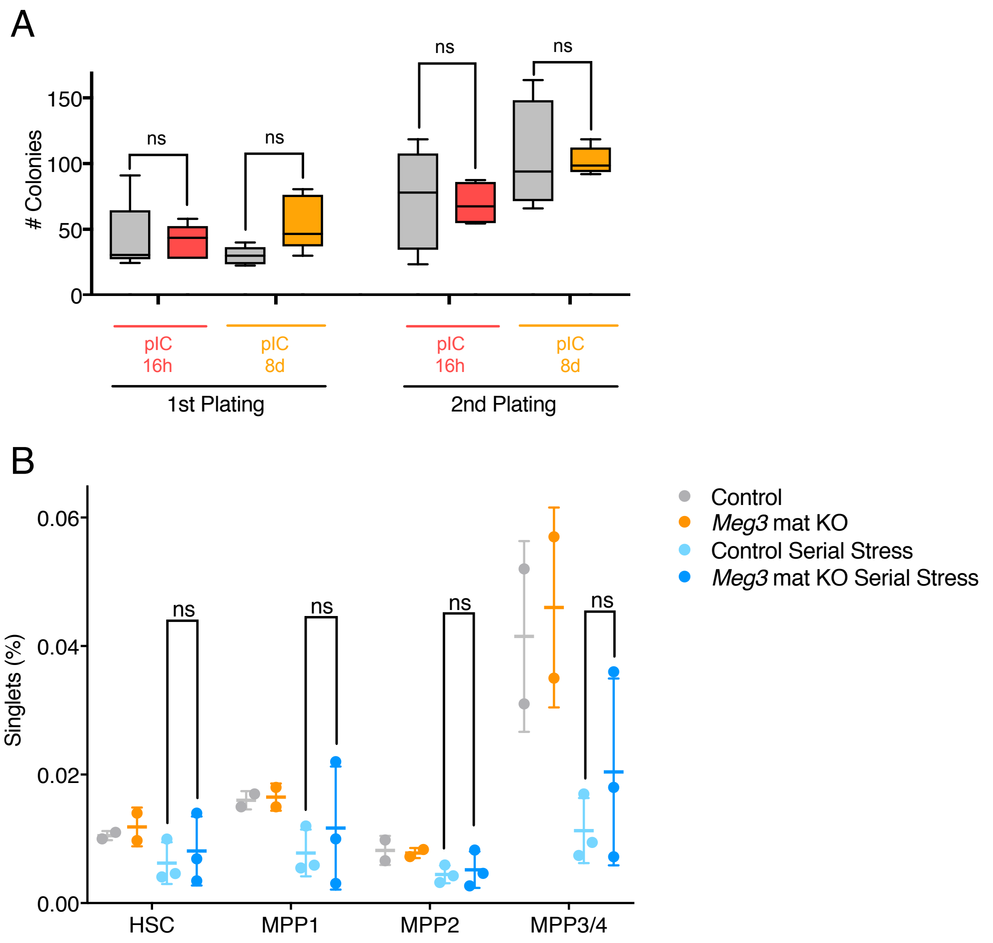

Supplementary Figure 4: (**A**) Functional analysis by serial CFU assays. n=5, one-way ANOVA. ns: not significant. n indicates number of biological replicates; 2 independent experiments. (**B**) HSCs, MPP1, MPP2, and MPP3/4 frequencies determined by flow cytometry analysis. Percentage of BM HSC-MPPs are represented. n=2-3, unpaired student's t-test. ns: not significant. n indicates number of biological replicates; 1 independent experiment.
